# Correlation between clinical parameters of crown and gingival morphology of anterior teeth and periodontal biotypes

**DOI:** 10.1186/s12903-020-1040-x

**Published:** 2020-02-19

**Authors:** Xiao-jie Yin, Bang-yan Wei, Xiu-ping Ke, Ting Zhang, Meng-yang Jiang, Xia-yan Luo, Hui-qiang Sun

**Affiliations:** 1 Department of Prosthodontics, School of Stomatology, Shandong University, Jinan, 250012 Shandong China; 2 Shandong Key Laboratory of Oral Tissue Regeneration & Shandong Engineering Laboratory for Dental Materials and Oral Tissue Regeneration, Jinan, Shandong China

**Keywords:** Periodontal biotype, Gingival morphology, Crown morphology, Digital models, Cutoff value

## Abstract

**Background:**

In this study, we conducted a quantitative analysis of the clinical parameters of crown and gingival morphology (CGM) of the maxillary anterior teeth (MAT). We also analyzed the correlation of these parameters with periodontal biotype (PB), with a view to providing objective standards for PB diagnosis.

**Methods:**

The three-dimensional (3D) maxillary digital models of 56 individuals were obtained using an intra-oral scanner. The following parameters were measured with the SpaceClaim software: gingival angle (GA), papilla width (PW), papilla height (PH), crown length (CL), crown width (CW), crown width/crown length ratio (CW/CL), bucco-lingual width of the crown (BLW), contact surface width (CSW), and contact surface height/crown length ratio (CS/CL). The PB were determined based on the transparency of the periodontal probe through the gingival sulcus. Independent factors influencing PB were analyzed by logistic regression, and the optimal cutoff values for the independent influencing factors were analyzed using receiver operating characteristic curves (ROC curves).

**Results:**

There was no significant difference in the parameters of CGM of the MAT at the left and right sides. The thick biotype accounted for 69.6%, and the parameters of GA, PW, PH, CW, CW/CL and CS/CL were significantly correlated with PB (*P* ≤ 0.2). GA (odds ratio (OR) = 1.206) and PW (OR = 5.048) were identified as independent predictive factors of PB, with areas under the ROC curve (AUC) of 0.807 and 0.881, respectively, and optimal cutoff values of 95.95° and 10.01 mm, respectively.

**Conclusion:**

The CGMs of the MAT at the left and right side are symmetrical. The thin biotype accounts for a small proportion, and GA and PW are independent influencing factors of PB. GA of 95.95° and PW of 10.01 mm are the optimal cutoff values for categorization of individuals as thick biotype. This indicates that when the GA and PW of the right maxillary central incisor are G ≥ 95.95° and ≥ 10.01 mm, respectively, there is a higher probability that these individuals will be categorized as thick biotype.

## Background

In 1989, Seibert and Lindhe [1] proposed the concept of PB, that is, the thickness of the bucco-lingual gingiva can be divided into thick and thin biotypes. The CGM coordination and the stability of the gingival margin differ between different PBs, which directly influences the esthetic effect of restoration and patient satisfaction. Furthermore, it has been proposed that PB has an important influence on the treatment effect and prognosis of periodontal surgery, plantation and orthodontics [2–4]. Therefore, correct classification of the PB is critically important in dental treatment.

In recent years, the correlation of PB with clinical parameters such as gingival thickness, crown morphology, and alveolar bone morphology has received increasing attention [5–12]. De Rouck et al. [5] measured intra-oral indexes including CW/CL, keratinized gingival width, and papilla height using calipers and a periodontal probe, and divided PBs into thin-scalloped biotype, thick-flat biotype and thick-scalloped biotype using the cluster analysis method. Stein et al. [10] conducted intra-oral measurements of keratinized gingival width and gingival thickness using a periodontal probe, measured the CW/CL and papilla height using image analysis software, and explored the correlation of parameters such as gingival thickness and CW/CL. However, the correlation of PBs with maxillary margin and papilla width, in addition to the independent influencing factors of PBs, remain to be clarified.

The use of calipers and periodontal probes to measure the clinical parameters of gingival morphology are subject to the disadvantages of inconvenience and low accuracy. The results obtained using image analysis software are inclined to be influenced by the position of the head although most reports do not include a unified stipulation of the spatial position of head [9, 13]. With the rapid development of computer technology, computer-aided design/computer-aided manufacturing (CAD/CAM) technology has been introduced for the esthetic restoration of anterior teeth. Lee et al. [14] analyzed the correlation between PB and the gingival papilla of the MAT using 3D digital models, while Wong et al. [15] explored the esthetic relationship between the incisal edge of the MAT and the upper border of the lower lip using 3D digital models.

In this study, we conducted a quantitative analysis of the clinical parameters of MAT, including GA, PW and PH, using an intra-oral scanner and SpaceClaim software, with a view to providing a more accurate reference for the computer-aided esthetic analysis and design of anterior teeth. The transparency of the periodontal probe through the gingival sulcus was used for periodontal biotyping, thereby exploring the correlation of the PB of the right maxillary central incisor with the clinical parameters of the gingiva and crown, and analyzing the cutoff value of independent influencing factors with the aim of providing objective standards for periodontal biotyping.

## Methods

### Study participants and inclusion criteria

From January 2018 to June 2018, 56 study participants (13 males and 43 females) were selected from the on-campus students and young nurses from the School of Stomatology of Shandong University (China). The participants were Han nationality and the average age was 23.6 ± 2.8 years. The following inclusion criteria were applied: 1) the MAT and the maxillary first molar on both sides were in orderly alignment and had no anodontia, interspersed diastema, wedge-shaped defect, dental caries, dental fillings or restorations; 2) healthy periodontal tissue: plaque index and gingival index ≤1, without obvious gingival recession or periodontal disease history; 3) no sleep bruxism history, no attrition of full dentition (attrition score ≤ 2); 4) normal or incomplete overbite and overjet; 5) normal occlusal curve; 6) no administration of gingival hyperplasia-related medicines within the latest 3 months; 7) no obvious gingival color pigmentation; and 8) age: 18–40 years.

### 3D digital model construction

**Fig. 1:**
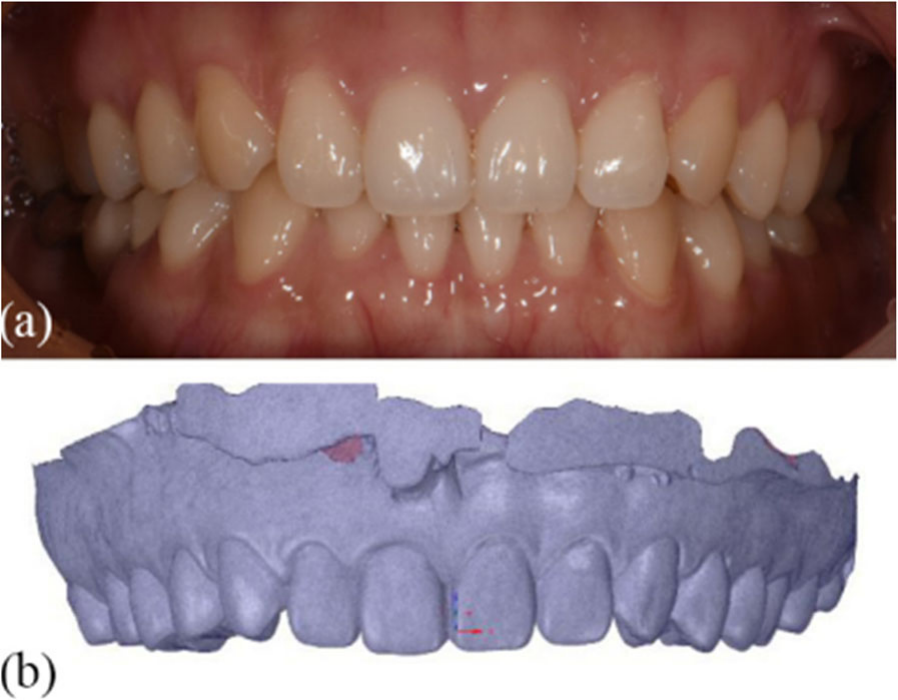
Intra-oral image and 3D digital models. **a** Intra-oral image (**b**) 3D digital model

The maxillary casts of the study participants were scanned using an intra-oral scanner and the STL format model files were numbered #1–#56. The *.STL files were imported into SpaceClaim software to generate 3D digital models (Fig. 1).

### Determination of the reference plane and esthetic landmarks

**Fig. 2:**
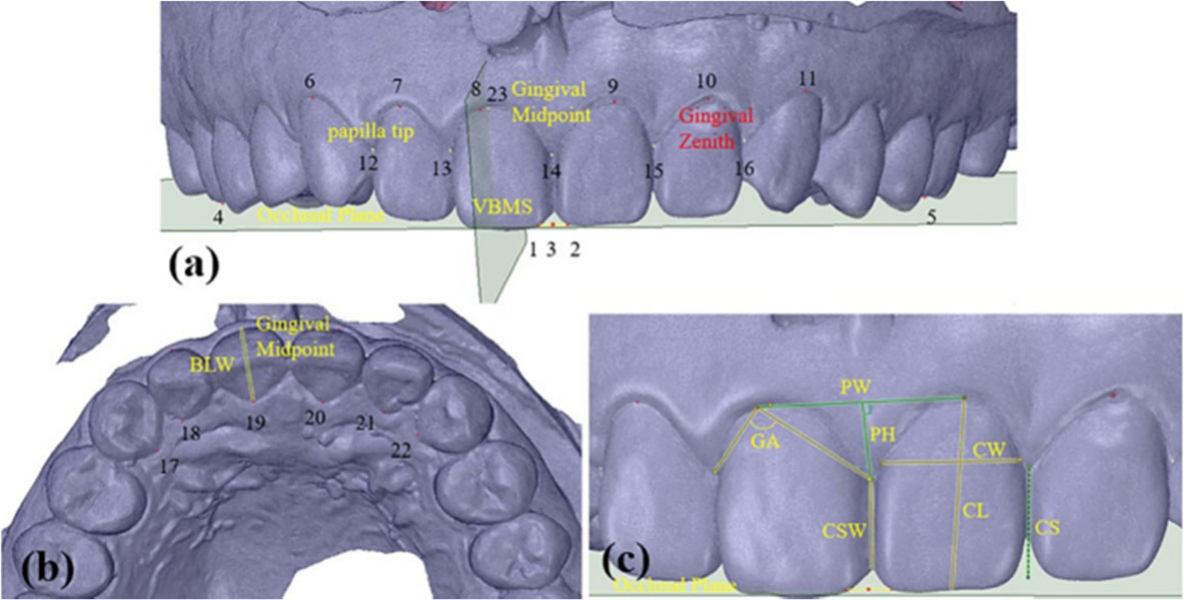
Esthetic-related landmarks (**a**) Landmarks 1 and 2, the mesio-incisal angle of the maxillary central incisors on both sides; Landmark 3, the midpoint of the mesio-incisal angle ligature between the maxillary central incisors on both sides; Landmarks 4 and 5, the mesio-buccal cusp tip of the maxillary right and left first molars; Landmarks 6–11, the gingival edge apical top of the MAT on the labial side; Landmark 12–16, the top of the gingival papilla of the MAT; Landmark 23, the midpoint of the right maxillary central incisors at the gingival edge midpoint. **b** Landmarks 17–22, the top at the direction of the gingival edge of the MAT on the palate side

The reference plane and esthetic landmarks were identified as previously described [16]. Briefly, for the description of the positional relationship between the maxillary dentition and gingival landmarks, an occlusal plane was selected as the horizontal reference plane, with 22 esthetic landmarks including the gingival zenith and the vertical bisected middle surface along the long axis of the clinical crown (VBMS). The intersection between the VBMS and gingival margin at the labial side was marked as the midpoint (Fig. 2).

### CGM index and measurement (accuracy: 0.01 mm)

CGM indexes were measured according to specific definitions as follows:

- Gingival angle (GA): the angle between the gingival zenith at the labial side and the top of the corresponding mesial and distal papilla [9] (Fig. 2c).
- Papilla width (PW): the distance between the gingival zeniths of the two adjacent teeth. Each value was then associated with the mesially positioned tooth (Fig. 2c).
- Papilla height (PH): the shortest distance from the top of the papilla to the segment PW [14]. The mesial PH was recorded for every tooth position (Fig. 2c).
- Crown length (CL): the distance from the gingival zenith to the midpoint of incisal edge (or dental cusp). (Fig. 2c).
- Crown width (CW): the distance between the approximal tooth surfaces was recorded at the border between the middle and cervical portions. (Fig. 2c).
- Bucco-lingual width of the crown (BLW): the distance from the gingival margin at the side of the palate side to the apex to the gingival midpoint on the labial side [9]. (Fig. 2b).
- Contact surface width (CSW): the distance between the contact areas of the most apical portion and the most incisal portion. The mesial CSW was recorded for every tooth position. (Fig. 2c).
- Contact surface height (CS): the shortest distance from the most apical point in the mesial contact area to the incisal edge [13]. The mesial contact area height was recorded for every tooth position, and CS/CL value was calculated (Fig. 2c).

All measurement data were obtained by the same clinical researcher. After an interval of 1 month, six samples were randomly selected from the master samples for re-measurement.

### Evaluation of PBs based on the transparency of the periodontal probe through the gingival sulcus

Study participants lay in the supine position on the treatment chair, with the occlusal plane perpendicular to the ground. After the area of the MAT was dried and a black background put into the mouth, the standard Williams periodontal probe (KPW, Shanghai Kangqiao Dental Instruments Factory, Shanghai, China) was placed into the sulcus at the midfacial aspect of the right maxillary central incisor. The periodontal probe was placed parallel to the long axis of the clinical crown at a probing depth of 1 mm (the probe would reach to the bottom of the gingival sulcus at a probing depth < 1 mm) [17]. A digital camera (Nikon D750, Nikon Corporation, Tokyo, Japan) equipped with a Microspur 105 mm lens (AF-S VR 105 mm f/2.8G IF-ED, Nikon Corporation) and a Microspur flashlight (Nikon R1C1, Nikon Corporation) was used to take photos in a standardized manner as follows: 1) unified shooting conditions with respect to factors such as light, background, and distance; 2) adoption of the same camera setting parameters; and 3) the teeth evaluated were placed at the center of photos, which should include around two natural teeth. All the examinations were completed by the same postgraduate student, and all photos were obtained by the same nurse.

**Fig. 3:**
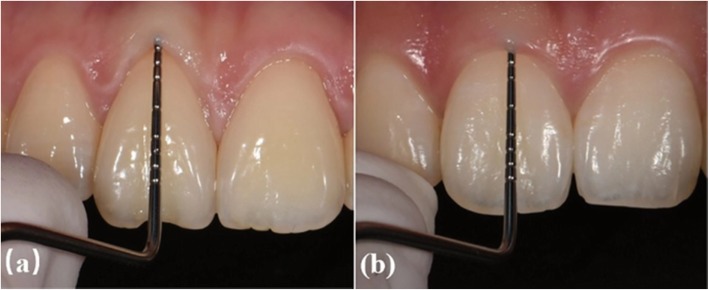
Periodontal biotype. **a** Probe visible through marginal tissue; thin biotype. **b** Probe not visible through marginal tissue; thick biotype

The photos of all experimental subjects were randomly placed into the PPT file (Microsoft). After training and alignment by examiners, three postgraduates independently conducted qualitative periodontal biotyping according to the following standards for PB classification [5, 10, 14, 18, 19]: 1) for thin biotypes, the probe was visible through the gingival tissue when placed within the gingival sulcus (Fig. 3a); 2) for thick biotypes, the probe was not visible through the gingival tissue when placed within the gingival sulcus (Fig. 3b).

After an interval of 2 weeks, the qualitative periodontal biotyping repeated independently, with the aim of lowering the bias in the first assessment. The determination of PBs for every study participant was based on consistent biotyping by two out of three examiners, with Kappa adopted to verify the reliability.

### Statistical analysis

For all the continuous variables (e.g., GA and PW), the test-retest reliability of the examiners was evaluated through the Pearson correlation coefficient test using SPSS Statistics 24.0 software (IBM, Chicago, USA), while the intra-examiner repeatability was evaluated with the Kappa test. In addition, the Shapiro–Wilks test was used to verify the normal distribution of data. The average values of clinical parameters were further analyzed by verifying that there was no significant difference in measurements of clinical parameters of the teeth at the same position on both sides of the mouth using the Wilcoxon signed-rank test and paired sample *t*-test. The normal distribution of data was confirmed, with measurement data expressed in mean ± standard deviation (SD). Analysis of variance (ANOVA) was used to evaluate differences between groups. Enumeration data were expressed as frequencies, and the chi-square test (*χ*^2^ test) was used to evaluate differences between groups. PB was assigned as a dependent variable, while the factors with ANOVA *P* ≤ 0.2 were assigned as independent variables. Forward: LR method was used for logistic regression to study the independent influence factors of PBs (inclusion equation standard 0.05, elimination standard 0.10). ROC curve analysis was repeated to evaluate the value of the factors that were finally entered into the logistic regression model for the diagnosis of PBs. *P-*values < 0.05 were considered to indicate statistical significance.

## Results

Test-retest reliability analysis showed relatively high reliability of all indexes (*r* ≥ 0.916, *P* < 0.001), except the Pearson correlation coefficient in the PH associated with the mesial aspect of the maxillary canines (*r* = 0.657, *P* < 0.001), indicating the repeatability of the data. The average Kappa coefficient of the transparency of the periodontal probe through the gingival sulcus was 0.733 (*P* < 0.001), indicating the high degree of reliability of the evaluation method. The Wilcoxon signed-rank test and paired sample *t*-test. Results showed that there were no significant differences in the measurements of clinical parameters such as GA and PW for the teeth at the same location and interdental position on both sides of the mouth (*P* ≥ 0.069).

The CGM characteristics of the MAT of the study participants are as shown in Table 1.

**Table 1:** The CGM characteristics of MAT

Factor	Central incisor	Lateral incisor	Canine
	Mean ± SD	Mean ± SD	Mean ± SD
GA(°)	98.19 ± 7.69	96.24 ± 10.03	89.45 ± 6.63
PW (mm)	10.05 ± 0.79	7.83 ± 0.60	7.97 ± 0.65
PH (mm)	3.65 ± 0.59	3.37 ± 0.53	3.28 ± 0.57
CL (mm)	9.53 ± 0.77	8.26 ± 0.77	9.09 ± 0.79
CW (mm)	7.51 ± 0.62	5.84 ± 0.48	6.64 ± 0.52
CW/CL	0.791 ± 0.077	0.713 ± 0.081	0.735 ± 0.070
BLW (mm)	7.22 ± 0.53	6.56 ± 0.52	8.38 ± 0.48
CSW (mm)	4.39 ± 0.72	3.56 ± 0.56	2.62 ± 0.57
CS/CL	0.597 ± 0.069	0.623 ± 0.070	0.635 ± 0.057

The periodontal biotyping of the study participants is shown in Table 2. Our results showed that among the thick biotype accounted for the largest proportion (69.6%) of the 56 study participants.

**Table 2:** The frequency distribution of PBs

	Male participants (n)	Female participants (n)	Total [n(%)]
Thin	1	16	17 (30.4)
Thick	12	27	39 (69.6)
Total	13	43	56 (100)

The characteristic parameters of periodontal biotypes of different genders are shown in Table 3. ANOVA test showed that there were statistical differences in periodontal biotypes between different genders.(*P* ≤ 0.2).

**Table 3:** Characteristics of periodontal biotypes in different genders

Index	Thin	Thick	*Χ*^*2*^*/F*	*P*
Gender			4.281	0.043

For different PBs, the CGM characteristics of the right maxillary central incisors are shown in Table 4. ANOVA showed that there were significant differences among the PBs in terms of in GA, PW, PH, CW, CW/CL and CS/CL (*P* ≤ 0.2), but no significant differences in terms of CL, BLW, and CSW (*P* > 0.2).

**Table 4:** The CGM characteristics of the right maxillary central incisors in different PBs

Index	Thin	Thick	*Χ*^*2*^*/F*	*P*
GA (°)	92.73 ± 6.21	101.68 ± 8.03	16.704	0.000
PW (mm)	9.43 ± 0.53	10.39 ± 0.61	31.955	0.000
PH (mm)	3.91 ± 0.58	3.53 ± 0.56	5.163	0.027
CL (mm)	9.69 ± 0.81	9.41 ± 0.81	1.428	0.237
CW (mm)	7.15 ± 0.58	7.67 ± 0.61	8.644	0.005
CW/CL	0.736 ± 0.065	0.819 ± 0.078	14.465	0.000
BLW (mm)	7.16 ± 0.53	7.25 ± 0.59	0.264	0.61
CSW (mm)	4.33 ± 0.70	4.50 ± 0.82	0.55	0.462
CS/CL	0.575 ± 0.074	0.611 ± 0.074	2.772	0.102

Table 5 presents the logistic regression results of multiple PB factors, showing that GA (OR = 1.206, *P* = 0.016) and PW (OR = 5.048, *P* = 0.002) were the independent influencing factors of PB. The logistic regression model was used to re-categorize PBs, with a total accuracy of 85.7%, as shown in Table 6.

**Table 5:** Multi-factor logistic regression of PB

Influencing factor	*OR*	*P*	*OR95%CI*	
			Lower limit	Upper limit
GA	1.206	0.016	1.035	1.405
PW	5.048	0.002	2.705	83.710

**Table 6:** Logistic regression prediction categorization of PBs

Observed	Predicted		
	Thin	Thick	Percentage correct
Thin	12	5	70.6
Thick	3	36	92.3
Overall percentage			85.7

**Fig. 4:**
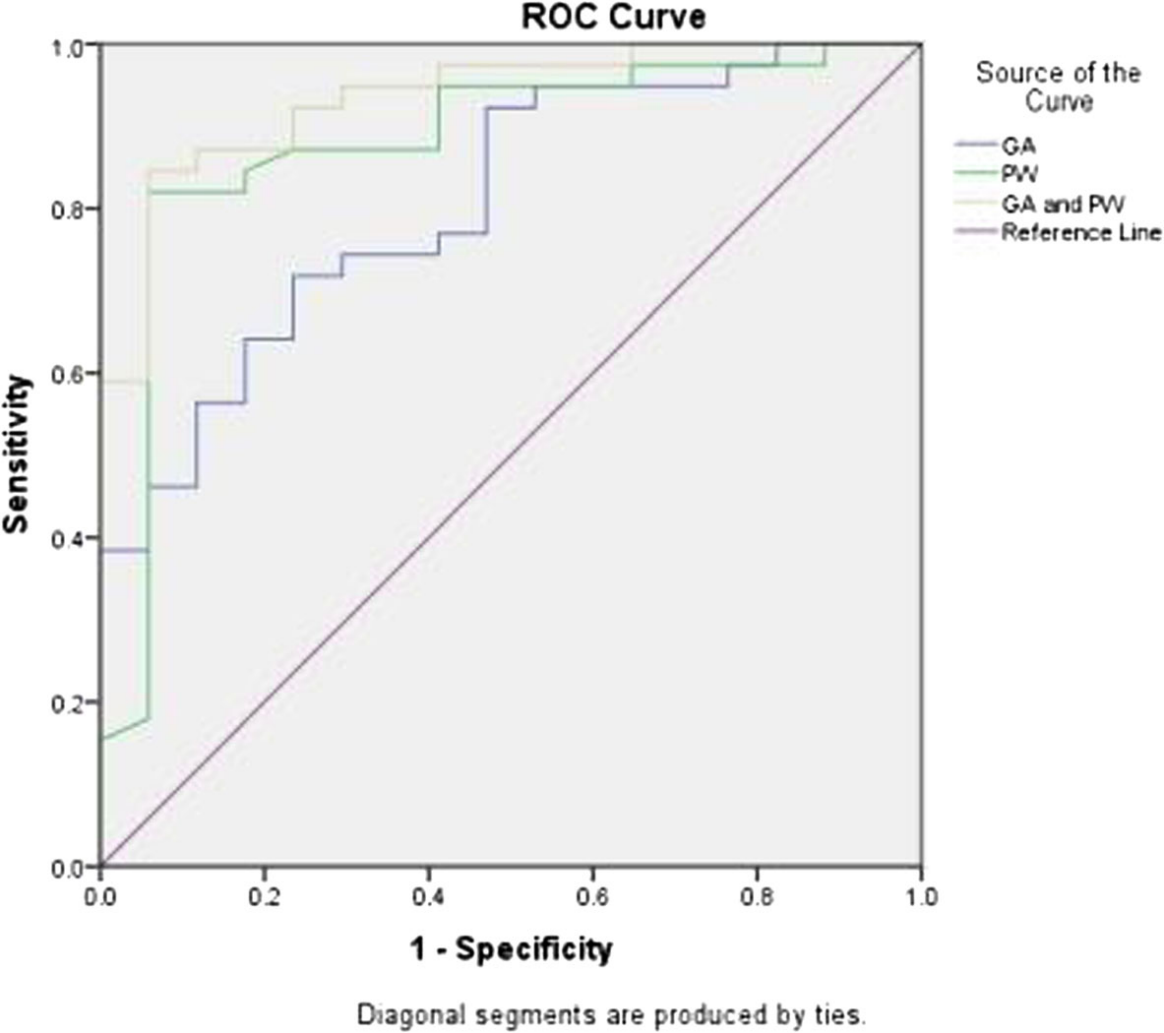
ROC curve plotting sensitivity and specificity values to predict thick biotype at various cutoff values of GA and PW

A ROC curve was generated with GA and PW as the test variables as shown in Fig. 4. The AUCs of GA and PW were 0.807 and 0.881, respectively, and the optimal cutoff values of GA and PW were 95.95° and 10.01 mm, respectively. The combined AUC of GA and PW was 0.935, which was larger than the singular AUC for GA and PW, showing that the combined diagnosis of GA and PW contributes to increasing the diagnostic efficiency of PBs. In other words, when the GA and PW of the right maxillary central incisor are 95.95° and 10.01 mm, respectively, the optimal cutoff value for categorizing study participants as thick biotype, as shown in Table 7.

**Table 7:** Diagnostic value of gingival edge angle and papilla width of PBs

Variables	AUC	95%CI	*P*	Sensitivity (%)	Specificity	Cutoff values
GA (°)	0.807	0.689–0.925	0.000	71.8	76.5	95.95
PW (mm)	0.881	0.774–0.988	0.000	82.1	94.1	10.01
GA and PW	0.935	0.871–0.999	0.000	0.846	0.941	0.76

CI: Confidence Interval

## Discussion

In clinical practice, different PBs may react differently to inflammation and various types of dental treatment. Accurate pre-treatment diagnosis of the PB of patients is necessary to obtain the ideal effect. In this study, we conducted a quantitative analysis of the CGM morphology in terms of the clinical parameters of upper anterior teeth using 3D digital models with the aim of providing an accurate reference for the esthetic analysis and computer-aided design of anterior teeth, determining the cutoff value of gingiva and crown clinical parameters, and establishing clinical guidelines to offer quantitative guidance for periodontal biotyping.

Among the 56 participants included in this study, the thick biotype accounted for the largest proportion (69.6%), while thin biotype accounted for only 30.4%. Furthermore, there was statistically significant difference in PBs between males and females, as shown in Table 3, which is consistent with the results reported by De Rouck et al. [5]. However, Lee et al. [14] found that sex had no significant influence on PBs, with the thin biotype accounting for 21.8% of the individuals evaluated. In contrast, Frost et al. [18] reported that the thin biotype accounted for only 7% of their study participants. It can be speculated that sample size and ethnic differences may be the major factors contributing to the inconsistency in these results.

The contour of the gingival margin is determined by parameters such as the gingival angle, papilla width, and papilla height. The GA averages of the maxillary central incisor, lateral incisor and canines of all study participants were 98.19 ± 7.69°, 96.24 ± 10.03° and 89.45 ± 6.63°, respectively. However, Olsson et al. [9] reported GAs for the maxillary central incisor, lateral incisor and canines of 86.60°, 82.80° and 80.29°, respectively. Differences in measurement methods may account for these inconsistencies. Olsson et al. [9] determined GA with the cosine function using intra-oral images to generated 3D digital models. This is a simple, convenient and accurate method of reflecting the spatial positional relation of the teeth and gingiva. The logistic regression model used in this study showed that GA (*P* = 0.016, OR = 1.206) is an independent influencing factors of PB. Our study also showed that the central incisor GAs of the thin and thick biotypes were 92.73 ± 6.21° and 101.68 ± 8.03°, respectively. These results are consistent with those reported by Olsson [9] and Zhou Zhixuan et al. [4], suggesting that the GA of the thin biotype is smaller and the gingival margin more curved than that of the thick biotype.

The morphology of the gingival papilla is a major evaluation index used in various current anterior teeth esthetic evaluation systems. The present study showed that the PWs of the maxillary central incisor, lateral incisor and canines in all the study participants were 10.05 ± 0.79 mm, 7.83 ± 0.60 mm and 7.97 ± 0.65 mm, respectively, which is consistent with the findings of Zhou Zhixuan et al. [4]. The logistic regression model of the right maxillary central incisor indicates that PW has a significant influence on PB (*P* = 0.002, OR = 5.048), making the gingival papilla of the maxillary central incisor of the thin biotype narrower, although there are few studies on the correlation between PB and PW.

Olsson et al. [9] proposed that the PHs for the maxillary central incisor, lateral incisor and canines were 4.16 mm, 4.02 mm and 4.21 mm, respectively, although the results of our study revealed values of 3.65 ± 0.59 mm, 3.37 ± 0.54 mm, 3.28 ± 0.57 mm, respectively. This disparity may be attributed to the differences in study participants and measurement methods. In addition, ANOVA showed a significant difference (*P* = 0.027) between PH and PB, while logistic multi-factor regression analysis suggested that PH is not an independent influencing factor of PB. De Rouck et al. [5] also reported a significant difference in PH between PBs, while Olsson [9] and Stein [10] et al. claimed that there was no obvious correlation between gingival thickness and PH. Lee et al. [14] found that the sum of five gingival papilla heights of the MAT larger than 24 mm was the identification standard for the thin biotype, and PB had no obvious correlation with the papillary height between two central incisors. The disparity in measurement methods and periodontal biotyping methods may be the major reasons for these differences.

Using image analysis software to calculate CW/CL, Stein et al. [10] found that CW/CL and PB were closely related, and therefore could be used to represent the predictive index for gingival thickness. In this study, ANOVA showed a significance difference between CW/CL and PB(*P* < 0.001), although in logistic regression model, CW/CL was eliminated from the regression equation, indicating that it is not an independent influencing factor of PB. This finding is consistent with those of Olsson [9] and Eger [20] and may be related to the difficulty in determining the most appropriate reference points, because CL is subject to the influence of attachment loss, gingival inflammation and incisal attrition, while CW is subject to the influence of the gingival papilla [9]. Moreover, differences in ethnicity and region may lead to different crown morphologies.

By measuring casts, Olsson [9] reported BLWs of the maxillary central incisor, lateral incisor and canines of 7.33 ± 0.56 mm, 6.51 ± 0.57 mm and 8.29 ± 0.65 mm, respectively, indicating a significant correlation between gingival thickness and BLW. According to our study, the BLWs of the maxillary central incisor, lateral incisor and canines are 7.22 ± 0.53 mm, 6.56 ± 0.52 mm, and 8.38 ± 0.48 mm, respectively, with no correlation between BLW and PB found. This discrepancy may be attributed to differences in the study participants and the PB diagnosis method.

Tarnow et al. [21] reported that the esthetic effect of the gingival papilla was associated with the position of the contact area. In this study, the CSWs of the maxillary central incisor, lateral incisor and canines were 4.39 ± 0.72 mm, 3.56 ± 0.56 mm, and 2.62 ± 0.57 mm, respectively, and the CS/CL values were 0.597 ± 0.069, 0.623 ± 0.070 and 0.635 ± 0.057, respectively. Moreover, compared with thin biotype, the contact surface of the thick biotype is wider, and the most apical portion of the contact area is closer to the gingival margin, although no significant differences were found between PBs. Gobbato et al. [13] categorized the maxillary central incisors, finding that the most apical portion of the contact area in the triangular group was closer to the incisal edge, while that in the square group was closer to the gingival margin.

Most previous studies have focused on the correlation of PB with the morphology of the soft and hard tissues [5–8, 10–12, 17, 19, 22–24], with little investigation of the influence of independent factors on PB diagnosis efficiency. The logistic regression results of our study show that the right central incisor GA and PW are important predictive factors of PB, with the probability of a thick biotype diagnosis increasing 1.206 times for every 1° increase in GA, while the probability of a thick biotype diagnosis increases 5.048 times for every I mm increase in PW. This supports the hypothesis that “compared with thick biotype, the free gingival margin at the labial side of the thin biotype is more curved and the gingival papilla narrower” [5, 9, 25, 26]. We found that the GA, PW and combined AUC were 0.807, 0.881, and 0.935, respectively, indicating that the combination of GA and PW improve the diagnostic efficiency of PB. In this study, the GA and PW of the right maxillary central incisors of 95.95° and 10.01 mm, respectively, were identified as the optimal cutoff values to categorize individuals as thick biotype. This implies an increased probability of categorizing individuals as thick biotype when the GA and PW of the right maxillary central incisors are ≥95.95° and ≥ 10.01 mm, respectively. In an analysis of the relationship between gingival thickness and PB based on ROC curves, Frost et al. [19] failed to identify a suitable gingival thickness threshold for diagnosis a thick biotype.

This exploration of CGM parameters and their correlation with PB using 3D digital models is limited by the small sample size, uneven sex ratio, and single focus on the correlation of the right maxillary central incisor PB with CGM clinical parameters. Therefore, in future studies, it is necessary to expand the sample size, balance the sex ratio, and take into consideration the correlation of the periodontal biotypes at different teeth positions with CGM. In addition, the limited number of influencing factors included in this research may have ignored the influence of other factors on PB; therefore, future investigations should evaluate the influence of factors such as alveolar bone morphology, keratinized gingival width and gingival thickness to provide more powerful evidence for the diagnosis of PBs in the clinic. In addition, randomized controlled trials are required to verify the potential of GA and PW for improving the accuracy of PB diagnosis.

## Conclusions

With the occlusal plane as the reference, the CGM at both sides is symmetrical. The thin biotype accounts for a small proportion of cases; in which the free gingival margin at the labial side of the central incisor is more curved, and the gingival papilla narrower than those of the thick biotype. For a long-narrow crown, the bucco-lingual width of the crown is smaller, the contact surface is larger, and the most apical portion of the contact area is closer to the incisal edge. Moreover, GA and PW are independent influencing factors of the PB of the right maxillary central incisor. The GA and PW of the right maxillary central incisors of 95.95° and 10.01 mm, respectively, are the optimal cutoff values to categorize individuals as thick biotype. This implies an increased probability of categorizing individuals as thick biotype when the GA and PW of the right maxillary central incisors are ≥95.95° and ≥ 10.01 mm, respectively.

## Data Availability

The datasets used and/or analyzed during the current study are available from the corresponding author on reasonable request.

## Abbreviations

3D: Three-dimensional
AUC: Areas under the ROC curve
BLW: Bucco-lingual width of the crown
CAD/CAM: Computer-aided design/computer-aided manufacturing
CGM: Crown and gingival morphology
CL: Crown length
CS: Contact surface height
CS/CL: Contact surface height/crown length ratio
CSW: Contact surface width
CW: Crown width
CW/CL: Crown width/crown length ratio
GA: Gingival angle
MAT: Maxillary anterior teeth
OR: Odds ratio
PB: Periodontal biotype
PH: Papilla height
PW: Papilla width
ROC curve: Receiver operating characteristic curve
VBMS: Vertical bisected middle surface along the long axis of the clinical crown
